# Reproducibility of findings in modern PET neuroimaging: insight from the NRM2018 grand challenge

**DOI:** 10.1177/0271678X211015101

**Published:** 2021-05-17

**Authors:** Mattia Veronese, Gaia Rizzo, Martin Belzunce, Julia Schubert, Graham Searle, Alex Whittington, Ayla Mansur, Joel Dunn, Andrew Reader, Roger N Gunn, and the Grand Challenge Participants

**Affiliations:** 1 Centre for Neuroimaging Sciences, Institute of Psychiatry, Psychology and Neuroscience, King’s College London, London, UK; 2 Invicro LLC, London, UK; 3 School of Biomedical Engineering and Imaging Sciences, St Thomas’ Hospital, King’s College London, London, UK; 4 Department of Brain Sciences, Imperial College London, London, UK; 5 King's College London & Guy's and St. Thomas' PET Centre, London, UK

**Keywords:** PET, data analysis, data sharing, reproducibility crisis, “NRM2018 PET Grand Challenge”

## Abstract

The reproducibility of findings is a compelling methodological problem that the neuroimaging community is facing these days. The lack of standardized pipelines for image processing, quantification and statistics plays a major role in the variability and interpretation of results, even when the same data are analysed. This problem is well-known in MRI studies, where the indisputable value of the method has been complicated by a number of studies that produce discrepant results. However, any research domain with complex data and flexible analytical procedures can experience a similar lack of reproducibility. In this paper we investigate this issue for brain PET imaging. During the 2018 NeuroReceptor Mapping conference, the brain PET community was challenged with a computational contest involving a simulated neurotransmitter release experiment. Fourteen international teams analysed the same imaging dataset, for which the ground-truth was known. Despite a plurality of methods, the solutions were consistent across participants, although not identical. These results should create awareness that the increased sharing of PET data alone will only be one component of enhancing confidence in neuroimaging results and that it will be important to complement this with full details of the analysis pipelines and procedures that have been used to quantify data.

## Introduction

A growing amount of evidence shows a concerning lack of reproducibility of scientific results across social science and medical disciplines.^1–4^ Neuroimaging is no exception. The inability to replicate experimental results poses a serious threat to the advancement of knowledge, questioning the scientific method at its very core. Among the principal reasons behind this methodological crisis are the lack of standardized pipelines for processing complex data and the lack of a complete description of the adopted methodologies in scientific papers.

Despite many attempts by the neuroimaging community to contain the problem by giving free and open access to data, software and methods, the problem remains more compelling than ever. In a recent fMRI study,^5^ 70 research groups across the world were asked to analyse the same neuroimaging data. Each of them produced different results. However, obtaining such a disappointing outcome is not new to fMRI. In 2008, Emily Murphy in her proactive blog “What a dead salmon reminds us about fMRI analysis”, showed how statistical artefacts could be obtained from this type of data without appropriate correction for multiple comparisons.^6^ This provocative work was systematically investigated, proving how methodology is important in the interpretation of fMRI studies.^7,8^ However, the problem cannot be isolated to fMRI, as it is not a problem of data type. On the contrary, it is likely that any research domain with complex and flexible data analysis procedures will have similar irreproducibility.^9^

In this paper, we wanted to investigate the reproducibility of the results for brain PET imaging studies, highlighting the impact that different data processing techniques and statistical approaches have on the reproducibility of findings. Quantification of PET data consists of linking the radioactivity measured in the scanner to the functional processes of the chosen biological system under investigation. It is well known that accurate and precise quantification using a series of individually designed processing steps (e.g. image reconstruction, motion correction, definition of regions of interest, kinetic modelling, partial volume correction, image statistics) is a critical part of PET analysis. This set of steps is not unique and each one of them can be implemented in different ways depending on the tracer, experimental design and research context (see for example ^10^ about [^11^C]DASB brain PET imaging). Most PET centres around the world have developed and optimised their own analysis pipelines, including a mixture of in-house or independent software and have implemented different modelling choices for PET image processing and data quantification.^11^ As a result, several different methods and tools are available for PET image analysis.

Understanding the possible impact of this lack of standardization in PET data analysis has become one of the top priorities of the PET scientific community, which has identified data sharing as the main solution for reproducible neuroimaging,^12^ leveraging optimal science and maximally powered research.^13^ Theoretically, applying the same analytical methods to the same PET data should lead to the same results. In practice, this might not always be the case as there are no standard procedures for data pre-processing and modelling, and different analytical choices can lead to discrepancies when data are re-analysed by different users, in line with what was observed in the Nature survey in 2016.^14^

A degree of work on reproducibility of PET data analysis has historically always been performed with scientists from different PET imaging labs commonly sharing data and software to evaluate differences in quantification from the same data. Researchers also spend time in each other labs, trying to identify the best practice for data acquisition and analysis.^15^ The goal here was to explore this further in an open competition setting and publish the results for visibility within the community.

To this aim, we proposed the PET Grand Challenge at the 2018 NeuroReceptor Mapping conference (London, 9–12 July 2018). We offered the molecular imaging community an opportunity to apply different approaches to the same set of simulated data in order to assess the performance and consistency of PET pre-processing and modelling approaches on a common data set where the ground truth was known. The challenge was to identify the areas and magnitude of receptor binding changes in a simulated PET neurotransmitter release study, using the image processing, kinetic analysis and statistical methods of choice. The participants were given minimal information on the tracer and target under study, and only the existence of a reference region devoid of the target of interest (explicitly indicated) was made known. Fourteen groups took part in the challenge, each providing their solution. As the purpose of the challenge was *not* to assess and evaluate individual performances, but rather to investigate the consistency of the PET modelling community, all the results in this paper are discussed anonymously.

## Methods

### The challenge

The challenge focused on the problem of PET parametric mapping using a reference-tissue quantification strategy. The data were simulated to mimic a neurotransmitter release experiment in which 5 participants underwent two dynamic PET scans, before and after the administration of a pharmacological challenge capable of stimulating neurotransmitter release in certain areas of the brain. The goal of the challenge was to identify areas and magnitude of changes in the receptor availability, knowing nothing about the pharmacology of the ligand, the target of interest or the administered blocking compound.

The participants were provided with a somewhat simplified scenario as compared to real brain PET imaging studies, to narrow the degrees of freedom in the data quantification. For example, data were shared already in MNI space and free from motion artefacts. This was to minimise the effect of spatial processing, which is well known to have a significant impact on the variability of results.^16^ Similarly, no arterial blood input function data were simulated as there is a big variety across PET centres for blood data pre-processing and fitting methods.^17,18^ Tissue time-activity curves were generated to fit the mathematical assumptions of the full reference tissue model,^19,20^ including an ideal reference region devoid of any tracer specific binding. This last decision was made to place the quantification methods in the best performing conditions, avoiding the systemic bias that affects analysis with suboptimal reference regions.^21^ The participants were asked to quantify the dynamic data to derive non-displaceable binding potential (BP_ND_) maps for each scan and to identify at the voxel level the areas of specific binding change after the pharmacological challenge. Each participant provided BP_ND_ maps for the 10 scans (all the 5 subjects, pre- and post-pharmacological challenge) and a summary displacement map containing the areas associated with a significant reduction of tracer binding. All these details were fully disclosed with the grand challenge participants on the website where they downloaded the data.

### PET grand challenge dataset: generation of the ground truth kinetic parameters

Five healthy subjects from an in-house existing dataset (NeurOimaging DatabasE (NODE), https://www.maudsleybrc.nihr.ac.uk/research/precision-psychiatry/neuroimaging/neuroimaging-database-node/) of [^11^C]Ro15-4513 PET scans were randomly chosen to generate the baseline data for the challenge. All the studies included in the NODE received ethical approval, required informed written consent from all participants, and were conducted according to the Declaration of Helsinki. Real data were used as starting point for the simulations rather than arbitrarily generated parametric maps to account for the spatial biological covariance of the brain tissues in a healthy brain volume.

An unconstrained reversible two tissue compartmental model (2TCM) was used for the quantification of the data voxel-wise. Blood data were measured accordingly to [^11^C]Ro15-4513 acquisition protocol^22^ and analysed using the Multiblood process^23^ (MATLAB code available at https://github.com/MatteoTonietto/MultiBlood). The 2TCM model was solved using the Variational Bayesian method^24^ to derive a parametric map for each individual rate constant and reach a satisfying level of homogeneity and a limited outliers percentage on the final parametric maps (*first level analysis*). Individual rate constants ( $K_{1},\;k_{2},\;k_{3}\;$ and $k_{4}$ ) and fractional blood volume ( $V_{B}$ ) were freely estimated.

A constrained 2TCM was hence used to re-quantify the data, using the following constraints to fulfil the Full Reference Tissue Model requirements^19^ (*second level analysis*):

- Cerebellum was artificially constrained to be the reference region (by setting 2TCM micro-parameter $k_{3}$ = 0 in all cerebellum voxels, as identified by the CIC atlas^25^)
- The 2TCM microparameter $k_{4}$ ( $=k_{\mathrm{off}}$ ) and $V_{B}$ were fixed to the whole-brain average values obtained in the first level analysis (after elimination of outliers, i.e. negative and unreliable estimates with a coefficient of variation higher than 100%).
- The tracer non-displaceable distribution volume $V_{ND}$ ( $=K_{1}/k_{2}$ ) was fixed to the average tracer total distribution volume $V_{T}$ estimates obtained in the cerebellum in the first level analysis (after outlier removal, defined as above).

In this second level analysis, PET data quantification for cerebellum, white matter and brainstem was performed with a simpler reversible one-tissue compartmental model, with both $V_{B}$ and $V_{ND}$ constrained across all the voxels of these regions to the same values obtained in the first level analysis as described above.

The resulting parametric maps were spatially filtered with local smoothing to eliminate possible outliers, using a median filter with a 4 mm kernel. Then the parametric maps were deconvolved in individual space to derive high resolution (i.e. 1 × 1 × 1 mm size) microparameter maps. Finally, the high-resolution manipulated parametric maps were normalised to the MNI space, to be then used as ground truth for data simulation.

### PET grand challenge dataset: Generation of the areas of displacement

**Figure 1.**
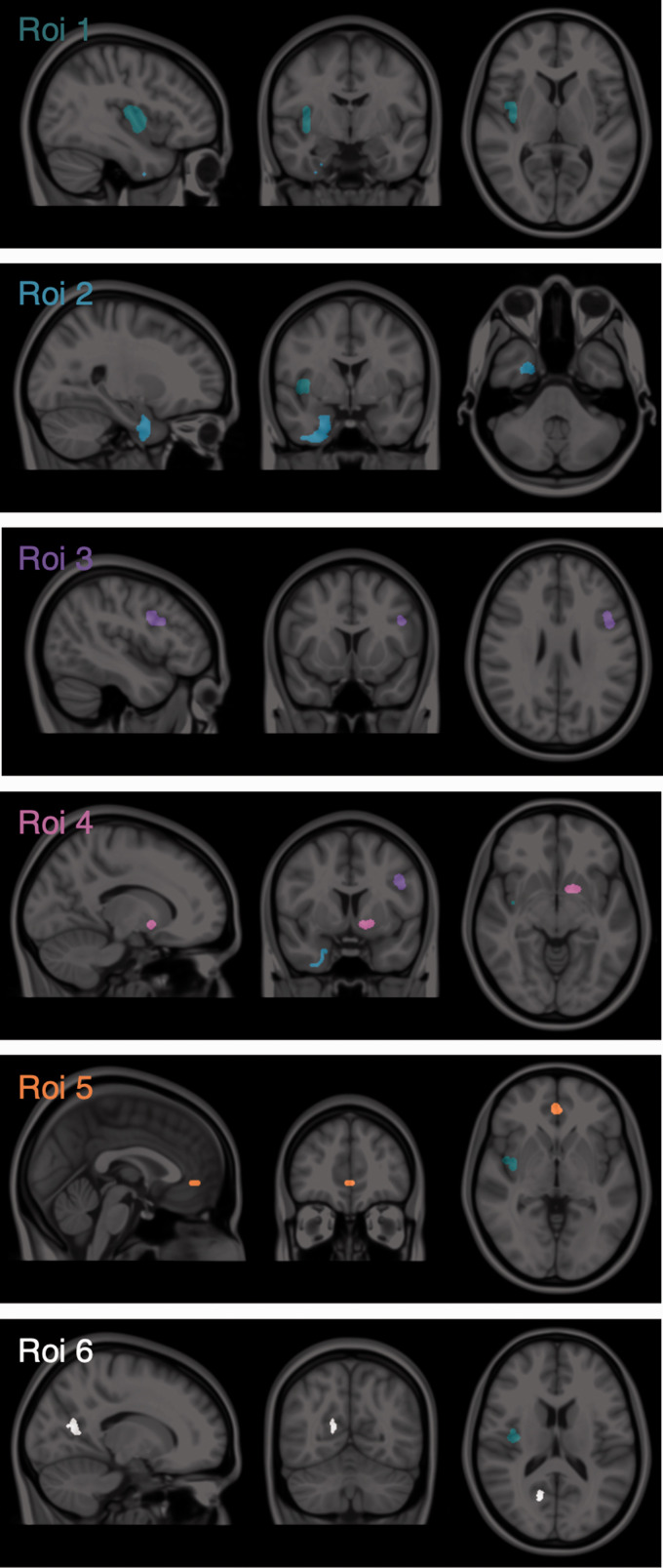
Simulated regions of tracer displacement. ROI 1 (green) ΔBP_ND_ = 27%, size = 2,555 mm^3^, Baseline BP_ND_ = 1.39. ROI 2 (blue) ΔBP_ND_ = 27%, size = 2,275 mm^3^, Baseline BP_ND_ = 1.65. ROI 3 (purple) ΔBP_ND_ = 21%, size = 1,152 mm^3^, Baseline BP_ND_ = 0.78. ROI 4 (pink) ΔBP_ND_ = 18%, size = 493 mm^3^, Baseline BP_ND_ = 1.16. ROI 5 (orange) ΔBP_ND_ = 18%, size = 343 mm^3^, Baseline BP_ND_ = 2.02. ROI 6 (white) ΔBP_ND_ = 18%, size = 418 mm^3^, Baseline BP_ND_ = 0.67.

Six regions of interest (ROIs) were chosen to test sensitivity and specificity of detection of tracer displacement for each methodology in a graded manner along two axes, with ROI size ranging from over 2,500 mm^3^ to under 400 mm^3^ and simulated displacement level ranging from 27% to 18% (Figure 1). ROIs were hand-drawn by the same user on the MNI152 template using ITK-SNAP^26^ (www.itksnap.org) with the following guidelines: 1) each ROI was one connected element, 2) ROIs were delineated so as not to exactly follow obvious anatomical structures or be geometric shapes (as these could bias the results towards certain methodologies) and 3) ROIs had to be spread across the whole cerebrum, rather than be localised in one particular brain hemisphere or lobe. A summary of the region characteristics are reported in Table 1.

**Table 1.** Regions of displacement.

ROI #	Region size (mm^3^)	Baseline BP_ND_ mean ± sd [min, max]		Displacement (%)
ROI 1	2555	1.39 ± 0.24 [1.09, 1.71]		27%
ROI 2	2275	1.65 ± 0.29 [1.20, 1.94]		27%
ROI 3	1152	0.78 ± 0.32 [0.46, 1.17]		21%
ROI 4	493	1.16 ± 0.66 [0.31, 2.06]		18%
ROI 5	343	2.02 ± 0.39 [1.49, 2.56]		18%
ROI 6	418	0.67 ± 0.23 [0.34, 0.93]		18%

### PET grand challenge dataset: data simulation

**Figure 2.**
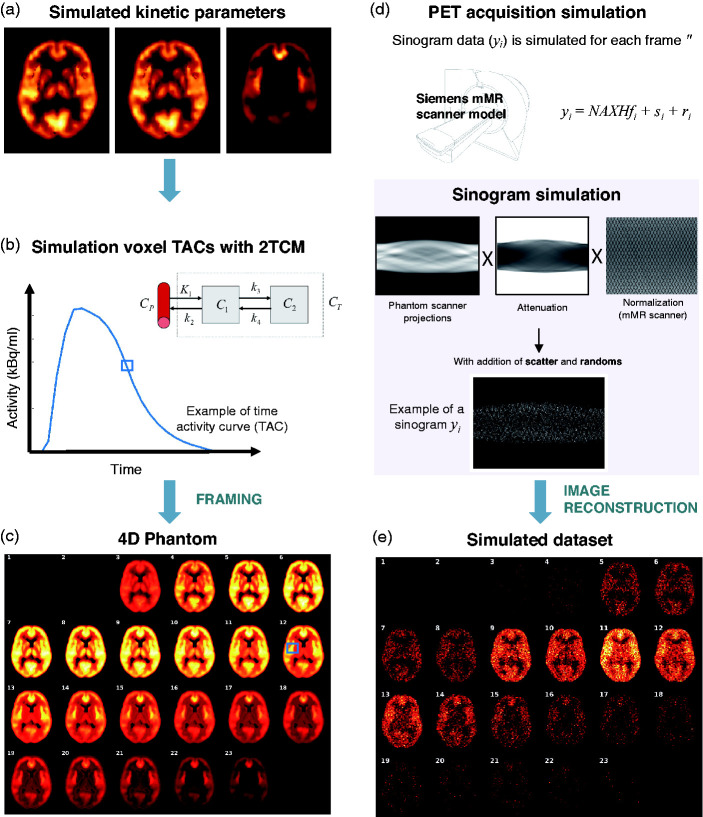
Data simulation pipeline. (a) Simulated kinetic parameters. (b) Simulation voxel TACs with 2TCM. (c) Generation of 4D Phantom. (d) PET acquisition simulation. (e) Simulated dataset. For a detailed description of each panel, please refer to main manuscript.

For each of the 5 subjects, two scans were simulated using the parametric maps previously described. First, we generated time-activity curves (TACs) using the 2TCM equations ^27^ for each 1 × 1 × 1 mm^3^ voxel of the parametric maps. The TACs were created with a temporal resolution of 1 second and included decay of the radiotracer using 20.34 min ^11^C half-life (Figure 2(a) and (b)).

Second, we created a 4D simulated digital phantom for each scan using the following time framing: 4 × 15 s, 4 × 60 s, 2 × 150 s, 10 × 300 s and 3 × 600 s, making a total of 23 frames per phantom. The phantoms had the same resolution as the parametric maps (1 × 1 × 1 mm^3^). A 4 D phantom example with the radiotracer distribution for each frame is shown in Figure 2(c), where the TAC for a given voxel is also available.

Third, the PET data were forward projected into sinogram space (Figure 2(d)) by first smoothing the image with a 2.5 mm full width at half maximum (FWHM) kernel and then applying a Siddon projector. A full 4 D PET noisy acquisition with a total of 3 × 10^8^ counts was simulated from each 4D phantom as follows: Poisson noise was simulated as described in^28^ where the normalization factors and the geometry of the Siemens mMR PET-MRI scanner were modelled. The spatial resolution of the scanner was not modelled as this was already included in the 4D phantoms (Figure 2(c)). Attenuation, random and scatter effects were also included. To compute the attenuation factors, we created an attenuation map for each phantom using the CT scan of the respective subject. Randoms and scatter accounted for 20% and 25% of the total counts, respectively.

Finally, each frame was individually reconstructed using the MLEM algorithm with 100 iterations, a 2.5 mm point-spread function and the standard mMR voxel size (2.09 × 2.09 × 2.03 mm^3^). The reconstructed images were corrected for tracer radioactivity decay and resampled into the original MNI space (Figure 2(e)). For the simulation and reconstruction, an in-house reconstruction framework was used.^29^

### Evaluation of the solutions

The solutions provided by the participants were assessed considering two different performance criteria: 1) the capacity of returning the correct BP_ND_ values both in terms of single maps and in terms of magnitude of change in the displacement regions, and 2) the spatial identification of the simulated displaced regions.

For the first criterion, percentage root-mean-squared error (RMSE) was used as the performance metric. RMSE for the BP_ND_ estimates across all the voxels of the 10 parametric maps (RMSE BP_ND_) and for ΔBP_ND_ in the displacement regions (RMSE displacement) were calculated as

(1) $$\text{RMSE}=\frac{\sqrt{\sum_{i=1}^{N}\frac{{\left(y_{i}-\hat{y_{i}}\right)}^{2}}{N}}}{\bar{y}}*100$$

where $y_{i}$ represents the i-th estimate, $\hat{y_{i}}$ the corresponding i-th ground truth value, N the number of voxels in the map/displacement region, and $\bar{y}$ the mean of the ground truth values in the map/displacement region.

For the second criterion, i.e. the identification of the displacement regions, the Jaccard similarity index (J) was used to compare the areas of displacement provided by the participants ( A ) with the true simulated displacement areas ( $\hat{A}$ ):

(2) $$\text{J}=\frac{\left|A\cap \hat{A}\right|}{\left|AU\hat{A}\right|}=\;\frac{TP}{TP+FP+FN}$$

where TP corresponds to the true positive (i.e. number of voxels in the area of displacement correctly identified as such), FP corresponds to the false positive (i.e. number of voxels outside the simulated area of displacement that were erroneously identified as displaced voxels) and FN corresponds to false negative (i.e. number of voxels inside the simulated area of displacement that were erroneously identified as non-displaced voxels).

Given the variability of analysis settings used by the participants, both in terms of data quantification and statistical parametric mapping, exploratory analyses to understand possible associations between analysis choices and performance levels would have been very interesting. However, the small number of solutions (N = 14) and the particularities of the testing data impede a meaningful statistical analysis or the possibility to give a general interpretation beyond the tested scenario. We therefore decided to qualitatively investigate similarity among the top solutions both in term of quantification bias and statistical mapping.

The aim of this analysis was to understand which analysis components and methods were most critical to superior performance. Data smoothing (yes/no), type of quantification methods (Logan/SRTM/MRTM2, see results for more details), data weighting (yes/no) and partial volume correction (yes/no) were considered as descriptive variables for RMSE BP_ND_ and RMSE displacement. Statistical smoothing (yes/no), type of statistical testing (parametric/non-parametric) and multiple comparison corrections (yes/no) were also considered.

## Results

### Solutions

An overview of the quantification and statistical methods used by the fourteen groups who took part in the Grand Challenge is reported in Table 2, while the full list of analysis methods, correspondent abbreviations and software follows in Supplemental Tables 1 and 2.

**Table 2.** Overview of quantification and statistical methods.

Group	Kinetic modelling solution	Quantification	Software	Statistics	Software
1	Logan graphical approach (unweighted)	Reference region: Cerebellar cortex from FreeSurfer (v,6.0)Smoothing: NonePartial volume correction: frame by frame with PETPVCImplementation: Logan t* fixed as t^max^/3 i.e. fixed at 30 minutes after injection	In-house MATLAB code	Smoothing: None Type: non-parametric Description: Thresholding based on visual inspection Multiple comparison correction: FWE	In-house code
2	MRTM2 (unweighted)	Reference region: Cerebellum from WFU Pickatlas, with erosion to minimize potential signal contamination from brainstem and cortexSmoothing: Dynamic PET data smoothing with 5 mm FWHM Gaussian kernelPartial volume correction: NoneImplementation: *k*_2′_ parameter derived from fitting the MRTM model to anterior cingulate cortex PET data as high-binding region with cerebellum as reference region. MRTM2 BP_ND_ was estimated voxel-wise using a basis fitting approach	PMOD (v.3.711)	Smoothing: 8 mm Gaussian kernelType: parametricDescription: One-sample t-test on ΔBP_ND_ maps; contrast ΔBP_ND_ > 0; brain mask: MNI T1 brain (no skull).Multiple comparison correction: FWE	SPM8
3	Logan graphical approach plot and Likelihood Estimation in Graphical Analysis (weighted)	Reference region: Cerebellar grey matter from in-house atlas, thresholded at 50%Smoothing: NonePartial volume correction: NoneImplementation: Logan/LEGA t* = 37.5 min post-injection. The BP_ND_ images were then obtained as the average of the estimates from Logan and LEGA approaches.	In-house MATLAB code (BrainFit)	Smoothing: Gaussian filter with 2 mm FWHM kernel (BP_ND_<0 set to zero prior to smoothing)Type: parametricDescription: Paired t-test, with p < 0.001 uncorrected (voxel level)Multiple comparison correction: FWE	SPM8
4	MRTM2 (unweighted)	Reference region: Cerebellum from Desikan-Killiany atlasSmoothing: NonePartial volume correction: NoneImplementation: *k*_2′_ parameter derived from fitting the MRTM model to rostral anterior cingulate cortex PET data as high-binding region with cerebellum as reference region	PMOD (v.3.5)	Smoothing: 8 mm FWHM kernelType: parametricDescription: Paired t-test with p < 0.001 uncorrected (voxel level) and p < 0.001 FWE-corrected (cluster level)Multiple comparison correction: FWE	SPM12
5^a^	Logan graphical approach (unweighted)	Reference region: Whole cerebellum from MNI structural atlas, thresholded at 90%Smoothing: Dynamic PET data smoothing with 8 mm FWHM Gaussian kernel Partial volume correction: NoneImplementation: t* defined from preliminary region-based analysis of subcallosal area (Harvard-Oxford atlas) as high-binding region and fixed at 27 minutes after injection	In-house MATLAB code	Smoothing: 5 mm variance smoothingType: non-parametric Description: paired t-test (N permutation = 10,000) and threshold-free cluster enhancementMultiple comparison correction: FWE	FSL - Randomise
6	SRTM (unweighted)	Reference Region: Whole cerebellum from CIC atlasSmoothing: NonePartial Volume Correction: NoneImplementation: Basis-function method	MIAKAT (v4.2.6)	Smoothing: 8 mm FWHM Gaussian kernelType: parametricDescription: Paired t-test (p < 0.005), cluster threshold = 30Multiple comparison correction: None	SPM12
7	Logan graphical approach (unweighted)	Reference region: Cerebellum from the CVS-atlas in FreeSurfer (v.5.3)Smoothing: Dynamic PET data smoothing with 6 mm FWHM Gaussian kernelPartial volume correction: NoneImplementation: *k*_2′_ parameter derived from fitting the MRTM model to Nucleus accumbens PET data as high-binding region with cerebellum as reference region. Logan t* fixed to be the last 12 time points	In-house software	Smoothing: NoneType: parametricDescription: Paired t-test (p < 0.01)Multiple comparison correction: Monte-Carlo simulation with 1,000 iterations	FreeSurfer v.5.3 and 3dClustSim (AFNI v. 16.2.1, July 2016)
8	Logan graphical approach (unweighted)	Reference region: Cerebellar grey matter using in-house manually defined brain atlasSmoothing: NonePartial volume correction: NoneImplementation: Logan t* fixed at 10 minutes after injection	In-house MATLAB code	Smoothing: None Type: parametricDescription: one-tailed homoscedastic t-test (p < 0.05), no cluster size thresholdMultiple comparison correction: none	In-house MATLAB code
9	SRTM (weighted)	Reference region: Whole cerebellum from CIC atlasSmoothing: NonePartial volume correction: NoneImplementation: Basis-function method	MIAKAT (v4.2.6)	Smoothing: NoneType: parametricDescription: General Linear Model (p < 0.05).Multiple comparison correction: FWE	SPM12
10	SRTM and Logan graphical approach (weighted)	Reference region: Whole cerebellum from Hammers atlas and PVElab softwareSmoothing: None Partial volume correction: NoneImplementation: Optimisation of parametric methods on basis of SRTM regional estimates using whole cerebellum as reference tissue input	In-house MATLAB and IDL codes	Smoothing: 8 mm FWHM Gaussian kernelType: parametric Description: Paired t-test (p < 0.001) and cluster threshold = 25Multiple comparison correction: none	SPM8
11	MRTM2(unweighted)	Reference region: Bilateral cerebellar cortex from FreeSurfer v 6.0 segmentationSmoothing: Dynamic data smoothed with 4.6 mm isotropic 3 D Gaussian kernel in SPM12Partial volume correction: NoneImplementation: *k*_2'_ parameter derived from fitting the MRTM model to rostral anterior cingulate cortex PET data as high-binding region with cerebellum as reference region.	In house MATLAB code	Smoothing: variance smoothing with a sigma of 5 mm to the group variance map. A mask indicating voxels > 1.15 reference region AUC was used in voxel-wise analysis; voxels with T(BP_ND_)<2 were set to NaN.Type: non-parametricDescription: One-sample t-test (p < 0.01)Multiple comparison correction: none	FSL – Randomize and in house MATLAB code
12	MRTM2(weighted)	Reference region: Cerebellar cortex extracted from FreeSurfer, dilated and eroded in order to avoid capturing regions too close to the border. Smoothing: 4 D dataset smoothed with 10 mm FWHM Gaussian kernel Partial volume correction: NoneImplementation: *k*_2'_ parameter derived from fitting the MRTM model to cingulate cortex PET data as high-binding region with cerebellum as reference region.	In-house code (FastMap)	Smoothing: NoneType: parametricDescription: Mixed effects analysis with effective DOF of 70. Voxel-wise results were computed without clustering. Multiple comparison correction: Gaussian random fields correction with the number of resolution elements (resels) defined as the number of voxels divided by the downsampling ratio from Gaussian smoothing (1000).	In-house code (Second-level GLM analysis using JIP)
13	Logan graphical approach (weighted)	Reference region: Cerebellum from probabilistic FSL atlas, thresholded at 50%Smoothing: NonePartial volume correction: NoneImplementation: Logan t* fixed at 40 min after visual inspections of a couple of subjects (whole brain time activity curve). Reference region efflux rate constant (*k*_2'_) derived from SRTM fitted to the whole brain time activity curve and averaged between baseline and displaced exam for each subject	In-house MATLAB code	Smoothing: 5 mm FWHM Gaussian kernelType: non-parametricDescription: paired t-test (N permutation∼100, variance smoothing of the t-maps with a gaussian filter of 5 mm FWHM)Multiple comparison correction: FWE, Cluster forming threshold of 5 voxels with a significant level of 0.05 after FWE correction	SnPM13
14	SRTM (weighted)	Reference region: Cerebellar grey matter using PVElab software Smoothing: NonePartial volume correction: NoneImplementation: Basis-function method (N = 30)	In-house MATLAB code	Smoothing: 8 mm FWHM Gaussian kernelType: parametricDescription: paired t-test (p < 0.001) and cluster threshold = 50Multiple comparison correction: none	SPM12

FWHM: full width at half maximum; FWE: family-wise error.

^a^ Winning solution of the PET Grand Challenge contest.

Note: For the list of software and analysis methods please refer to Supplemental Tables 1 and 2.

In terms of quantification methods, Logan (6/14),^30^ SRTM (4/14) solved with a basis function implementation ^31^ and MRTM2 (4/14)^32^ were the kinetic analysis methods chosen to quantify BP_ND_ at the voxel level. Nevertheless, different implementation choices were made. These included different t* for the graphical methods, a different anatomical segmentation of the cerebellum time-activity curve to be used as the reference region (with and without grey matter masking) and different data weighting schemes for the fitting. Even the selection of a smoothing filter for denoising the data was not consistent across groups. In terms of software, the majority of the groups used in-house code (10/14 – mostly in MATLAB, 8/14) while the rest used PMOD® (2/14) or MIAKAT™ (2/14). Only one group applied partial volume correction after the generation of parametric maps.

**Figure 3.**
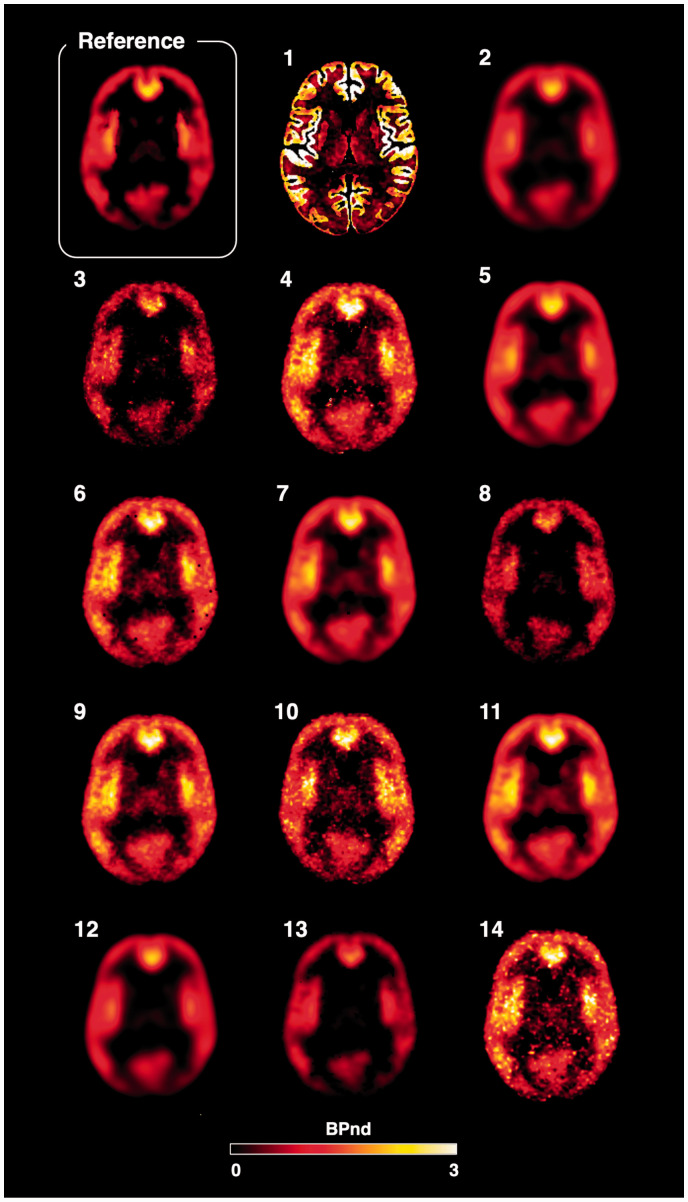
Parametric maps. Representative BP_ND_ maps for a baseline scan including the simulated reference map (top left corner) followed by the corresponding fourteen submitted solutions in matching order with Table 2.

A representative overview of the BP_ND_ parametric maps obtained from the participants is reported in Figure 3. Qualitatively, all the methods preserved the tracer spatial distribution present in the simulated reference maps, although with different noise distributions: the majority of the maps (7/14) had similar noise distribution to the simulated dynamic PET data (Figure 2(e)), while the others presented a smoothed appearance probably due to the use of denoising filters.

**Figure 4.**
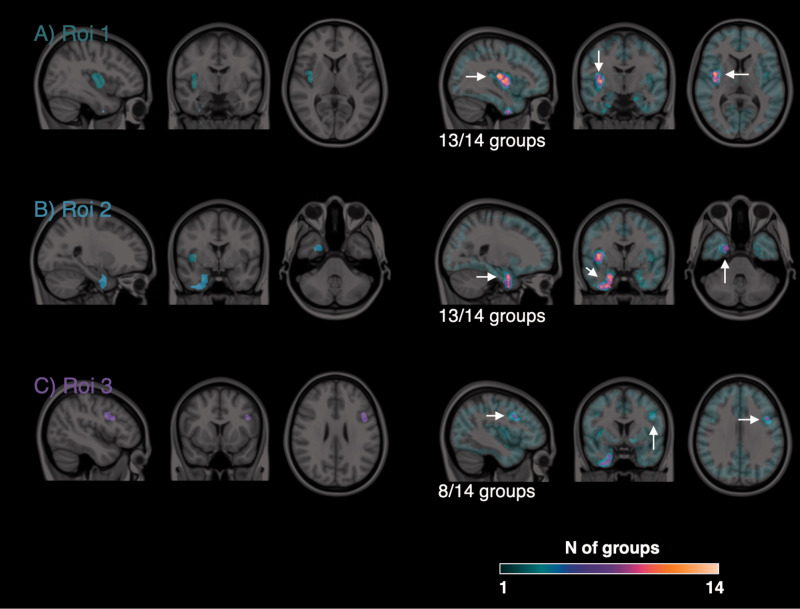
Identification of the areas of tracer displacement. Left images: Simulated regions of tracer displacement overlaid on structural MRI. Right images: common areas of displacement identified by participants. Colour intensity refers to the number of groups who identified a given voxel as part of an area of tracer displacement.

Similarly to the quantification, statistical analysis methods varied across submitted solutions in all possible settings: data smoothing kernel, parametric vs non-parametric analysis, cluster size and uncorrected significant threshold, multiple comparison correction and statistical software (Table 2). Nevertheless, the identification of the displaced ROIs was quite consistent. Thirteen out of 14 groups were able to identify significant clusters in ROI 1 and 2, the biggest areas with the greatest displacement (Figure 4(a) and (b)). Only 8 out of 14 groups were able to identify a significant cluster in ROI 3, which was a medium size region simulated with intermediate displacement (Figure 4(c)). The remaining regions (small size, low displacement) were consistently undetected by 13 out of 14 groups. The groups also performed very similarly in terms of false positive voxels (i.e. voxels in which the simulated PET did not change, but classified as displacement voxels by the participants), which represented a relatively small fraction of the true positive voxels (mean ± SD: 17% ± 23%).

### Performance

**Figure 5.**
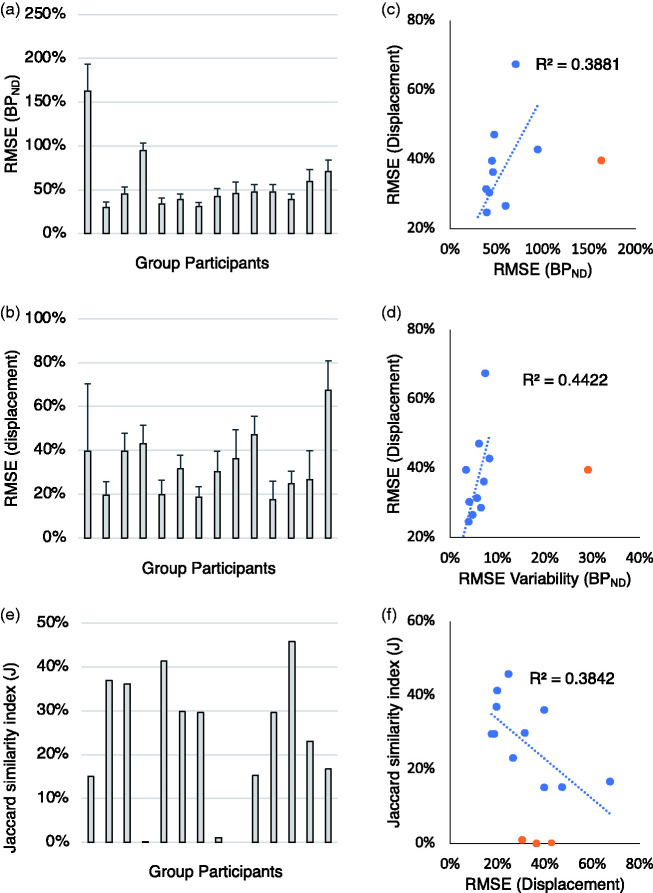
Method performances. (a–d) Percentage RMSE for baseline BP_ND_ estimates (RMSE BP_ND_) and for ΔBP_ND_ (RMSE displacement) as a function of the group participants and cross-correlation. (e–f) J similarity coefficients as a function of the group participants and cross-correlation with RMSE displacement. Blue circles refer to individual data value. Orange circles refer to outliers. For each bar graph, the order of solutions is presented in matching order with Table 2.

An overview of the group performances is reported in Figure 5. RMSE BP_ND_ across all the PET simulated maps ranged from 30%±6% (best performing solution) to 163%±31% (worst performing solution). These numbers reflect quantification error in the estimation of parametric BP_ND_ maps on average across the 10 scans. The average performance across groups led to a RMSE equal to 56%±37% (Figure 5(a)). The RMSE displacement ranged from 17%±5% (best performing solution) to 67%±21% (worst performing solution). These numbers represent quantification error in the estimation of ΔBP_ND_ in the simulated displacement regions (normalising by the simulated displacement). The average performance across groups led to a RMSE equal to 33%±17% (Figure 5(b)). Despite the different levels of bias between BP_ND_ and ΔBP_ND_, the two variables were unsurprisingly found to be associated (Pearson’s r = 0.62), with lower RMSE BP_ND_ leading to lower RMSE displacement across all the 6 ROIs simulated in the challenge (Figure 5(c)). Group 1 results were outliers (bias >150%) and hence not included in the regression (reported as orange circle in the figure). Similarly, a higher variability for RMSE BP_ND_ estimates across the 10 scans was associated with higher RMSE displacement (Pearson’s r = 0.66, Figure 5(d). Group 1 results reported as orange circle and not included in the regression). This test reflects the idea that when bias cannot be avoided (ideal scenario) a constant bias across scans is more desirable when looking at longitudinal changes than a non-constant bias.

The Jaccard similarity index (J) summarises the performance in the identification of the regions of displacement, with the best performing solutions reaching 40% and the worst performing solutions around 1% (Figure 5(e)). As expected, a better RMSE displacement is associated with a better J coefficient (Pearson’s r = 0.62, p = 0.03, Figure 5(f)) but also a lower RMSE variability is associated with higher J coefficient (Pearson’s r = 0.66, p = 0.02). The three J values smaller than 1% are reported as orange circles and were not included in the regression.

### Analysis of best performance

We identified four solutions returning a RMSE displacement <20%. Interestingly there was no agreement on the quantification method (2 solutions applied MRTM2 and 2 solutions used Logan), settings (e.g. t* or *k*_2’_) or reference region segmentation. The only element that all the solutions had in common was the use of spatial filter to smooth the data prior to quantification (FWHM >5mm).

There were only two solutions with J similarity index > 40%, which implemented quite different strategies, one implementing parametric testing and Gaussian random fields for multiple comparison corrections, and another one implementing nonparametric testing based on permutation and FWE multiple comparison correction.

The winner of the competition was Dr. Daniel Albrecht (Table 2, #5) whose solution returned RMSE displacement equal to 17%±5% (mean±sd) and J similarity index equal to 41%, ranking on among the top solutions for both quantification and statistical performance.

### Analysis settings vs performance

Analysis of variance showed that RMSE BP_ND_ was significantly associated with the quantification settings (R^2^=0.76, p = 0.01), in particular with the implementation of smoothing (F = 21.5, p < 0.01) and the type of quantification method (F = 6.9, p = 0.02). Data weighting in the modelling was not significant. Similarly, RMSE displacement was linked to the same parameters although the model did not reach significance (R^2^ =0.64, p = 0.06). Note that partial volume correction was not included as a variable, given than only one group implemented it. On the contrary no statistical variables were associated to the Jaccard similarity index (R^2^ =0.21, p = 0.53) including the choice of statistical tests (parametric vs non-parametric, F = 0.69, p = 0.43), smoothing (F = 0.76, p = 0.41) or multiple correction comparison (F = 1.10, p = 0.32).

Consistent with our expectations,^33,34^ Logan-based BP_ND_ parametric maps were more biased than the rest of the solutions (Logan-based BP_ND_ bias (N = 5): –23% ± 22%; Non Logan BP_ND_ bias: –4% ± 23%). However, when the methods were compared in term ROI displacement identification, Jaccard similarity indexes were comparable (Logan-based J (N = 5): 26% ± 14%; Non Logan J: 22% ± 16%), providing evidence that similar bias was present in both baseline and challenge scans which was then attenuated in the assessment of change.

## Discussion

### Reproducibility

This paper investigates the reproducibility of findings in a PET neuroimaging study by looking at the consistency of results provided by 14 imaging groups who took part in the PET Grand Challenge during the NeuroReceptor Mapping (NRM) conference in London 2018. The purpose of the challenge was to identify and quantify the tracer binding displacement in a simulated PET neurotransmitter release study. Participants were blinded to both tracer and pharmacological challenge.

The solutions proposed by the participants were all very different in terms of data quantification and statistical analysis choices. Nevertheless, the parametric maps were visually very similar and could be clustered in groups with comparable spatial quality and noise content (Figure 3). Moreover, the areas of displacement identified by the participants were also very consistent across groups, with 3 out of 6 simulated displacement regions (i.e. the ROIs with biggest sizes and highest displacements) identified by the majority of the groups (>50%) and 3 out 6 regions (i.e. the ROIs with smallest sizes and lowest displacements) identified by none of the groups. These last regions were particularly challenging as the study was probably underpowered for their identification: a post-hoc analysis using the variability of provided estimates on these regions confirmed the statistical power to be 32%±13% (Paired t-test, N = 5, alpha = 0.05). The main take home message regarding the reproducibility of results in this context is that if these groups were to write a paper around their results, they would describe the same pharmacological effect on the PET tracer, despite adopting different methodological choices.

It is somehow surprising that beyond this good agreement between displacement maps, the quantification bias on tracer binding and on tracer displacement estimates were not as consistent. First, not only do different methods lead to different biases, but there are clear associations between quantification choices and analysis performance. It is not the purpose of this work to comment on each individual method applied in this challenge, because the particular conditions set in the simulation would not necessarily be generalizable beyond this dataset. But it is clear that controlling for data noise (in this case by spatially smoothing the dynamic data) and choosing a specific parametric mapping method led to different parameter bias for both baseline BP_ND_ and ΔBP_ND_ estimates. Secondly, the bias becomes irrelevant in the identification of the areas of tracer binding displacement if it remains constant across different scans in the same subject. This is a fundamental point for neuroimaging studies looking at changes across different groups and conditions. It highlights that controlling for a balance of both bias and variability is important in the context of the particular study design and experimental question under investigation. However, it is important to highlight that the simulation process did introduce a systematic quantification bias due to the presence of partial volume and other source of errors of the simulated PET scanner (Supplementary Figure 1). Reanalysis of noise-free simulated data with the same model that was used to generate the data returned BP_ND_ estimates that were on average 12% lower than the true values.

Quite surprisingly, there was no correlation between the parameters set for the statistical testing and the performance of the statistical mapping. This seems to be counter intuitive, given evidence from multiple studies indicating how critical this step of analysis can be.^35^ Different reasons could explain this result, including the small number of scans to investigate these effects, the type of metric (i.e. J similarity index) used for the statistical performance assessment, and the impossibility of testing the t-statistical maps. It is worth noting that statistical parametric mapping in PET is less common than in other imaging modalities like MRI. When possible, region-based analyses are preferable as they have the advantage of using less noisy data than the voxel-based analysis and more robust quantification methods.^36^

Moreover, a good neuroimaging statistical method needs to control for false positive results while being sensitive to true positive cases.^37^ By definition of conventional thresholds, false positive results for a statistical method should represent only 5% of the total positive cases. However only 4 out of 14 solutions were able to contain them below such a threshold, while the rest obtained an average false positive rate of approximately 34%. This could be explained by the fact that not all the groups (only 6/14) implemented a multiple comparison correction strategy. Note that the location of these false positive voxels was mainly surrounding the true displacement voxels (Figure 4), indicating a less conservative identification of displacement regions, although not exclusively.

### How to boost result reproducibility in PET data analysis

In recent years, data sharing has increasingly been recognized by the neuroimaging community as one of the best resources for leveraging optimal research, tackling the concerningly low reproducibility of many neuroimaging findings.^12,13^ Data sharing supports imaging research reproducibility in many ways: it enables data quality control across sites, it boosts statistical power by providing larger samples, and supports thriving multilateral collaborations. As a result, research councils and scientific journals are increasing the requirement for data sharing, so that it will be necessary to share the data along with the publications of the results. Platforms for neuroimaging data sharing are already available, but dedicated resources for brain PET imaging are in course of development (e.g. https://openneuro.org/pet is now working on a PET-dedicated section).

But sharing imaging data does not necessarily imply sharing data modelling and analysis methods. PET neuroimaging represents a special case, as PET images are very rarely used in their raw form (as counts measured in the scanner), and, in contrast to most other imaging modalities, need to undergo a series of pre-processing steps before being suitable for quantitative analysis. Theoretically, applying the same methodology to the same PET data should lead to the same results. In practice this might not always be the case. There are no standard procedures for data modelling and different choices for analytical settings can lead to discrepancies when data are re-analysed by different users. Therefore, sharing PET data implicitly creates the need for more efficient communication of the methodological details of the quantification process than what is typically available in published studies. In the absence of such information, it may not be possible to reproduce published results, even if the raw data were available. Unfortunately, a concise and exhaustive description of methodological details is lacking in many papers, especially those published in clinical journals. For this reason, the international guidelines on PET data sharing encourage the use of a standardized checklist to be filed with a manuscript not only to facilitate archiving and data sharing but also to understand, interpret, and reproduce published work.^12^ The results of this work strongly support the use of such a standardized checklist, as the number of possible methodological choices can lead to discrepant results that would be difficult to interpret otherwise.

Standardisation of methods could be seen as an additional solution to boost reproducibility of results, especially when sharing data. The concept is simple: same data input, same method of analysis, same outputs. Reality is always more complex than theory. First, standardisation of methods is complex in PET. Methods tend to be tailored to the experimental design, data acquisition protocol and scanner(s) characteristics, and therefore it becomes difficult to find a single best solution for all possible scenarios, even when considering the same tracer. Secondly, sharing methods requires standardisation of both the data input and the analysis software. Some efforts have been made to share PET data using a standardised PET-BIDS format (https://bids.neuroimaging.io/bep009),^38^ but for the latter much work is still needed. As a matter of fact, 12 out of the 14 solutions provided in this paper used different computer programs for the kinetic modelling, many of which were developed in house. Thirdly, when a new PET tracer is translated into human for the first time, its deployment is generally coupled with formal methodological validation work.^39^ This includes investigating the robustness of the analysis pipeline and the quantification model in terms of reproducibility of the kinetic parameter estimates with a test-retest study, their biological sensitivity and specificity to the target with pharmacological blocking studies, as well as assessing experimental time stability and statistical power. This validation allows space for multiple solutions without imposing any standardisation, and it is generally done before and separately from application studies, defining the space for the interpretation of results, their statistical validity and biological plausibility. This process has been the methodological pillar for PET imaging for many years, which has supported its use in clinical and experimental medicine.

It is important to emphasise that data analysis is not the only aspect that determines the reproducibility of scientific findings in PET experimental medicine studies. In the broader framework of reproducibility, other issues, such as small sample sizes and low power, the unknown aetiology of many neuropsychiatric conditions and the resultant possible underlying heterogeneity with respect to the systems and targets studied, as well as a publication bias against negative results with resulting pressure to give manuscripts a positive spin, may contribute as much or more to poor reproducibility.^40^

### Limitations

This work has several limitations. Differently from other imaging reproducibility studies,^5,16^ the NRM2018 PET Grand Challenge used simulated data rather than real measured data. This is important, because even though ground truth was available, the dataset did not necessarily reflect the exact spatio-temporal signal/noise patterns of real data and present a particularly favourable scenario in which several pre-processing steps (e.g. motion correction and image normalisation to MNI) were pre-accounted for. Similarly, a valid reference region was made available and no blood data were used: it is expected that differences between participants would have been much higher if blood data were involved, as there are even more differences among PET centres on how to deal with the parent fraction, metabolite-corrected input function and vascular correction. Future PET data analysis challenges could also consider blood-based quantification methods. Although many studies have compared these types of analysis methods with different radiotracers and applications (see for example ^41–44^) a systematic evaluation on their reproducibility between research centres is still missing.

Moreover, the participants did not have full domain knowledge (e.g. considered tracer, target of interest etc.), which may have been used to inform their analysis. In reality, when a new tracer is tested for the first time, blood input function methods are tested before exploring non-invasive solutions (i.e. reference tissue models, SUV or tissue ratio methods). Moreover, preclinical imaging studies are generally performed prior to clinical studies to give an idea about the specificity of the tracer to its biological targets, as well as to its bio-distribution. Although, in contrast, the participants did have certain advantages not available in the real world – explicit confirmation that 1) there was no motion in the data set, 2) all simulations were performed in a standard brain space obviating the need for non-linear registration of brains into a common space and 3) a true reference exists and its anatomical location.

In terms of performance, it is very difficult to generalise the results beyond the context of the competition. First of all, this grand challenge represents a particular case of a PET neurotransmitter release experiment, and further testing should be done before generalising these results to other tracers and other types of PET experiments. Second, the study might have been underpowered to detect the small regions of displacement, making their identification extremely difficult. Last, a limited subset of quantification methods was implemented, and only a fraction of the brain PET community took part in the challenge.

## Conclusion

This paper shows that the choice of analytical and statistical procedures can have a substantial effect on the variability of findings in PET imaging studies, similarly to other neuroimaging techniques. Even in a simulated PET dataset, in which the analysis choices are controlled and limited as compared to a real-life study, this flexibility can lead to different results.

In such respect the brain PET community should continue its effort to improve reproducibility of its science by working together towards common and agreed methodologies and sharing clear descriptions of these processes in publications. When the standardisation of analysis pipelines is not possible, open-free shared datasets like the one provided by the NRM2018 PET Grand Challenge could serve as a tool for individual research groups to benchmark the performance of their analysis methods.

**Table:** List of Grand Challenge Participants

Author	Affiliation
Daniel S. Albrecht	Athinoula A. Martinos Center for Biomedical Imaging, Department of Radiology, Massachusetts General Hospital, Harvard Medical School, Charlestown, Massachusetts (USA)
Joseph B Mandeville	Athinoula A. Martinos Center for Biomedical Imaging, Department of Radiology, Massachusetts General Hospital, Harvard Medical School, Charlestown, Massachusetts (USA)
Christin Y Sander	Athinoula A. Martinos Center for Biomedical Imaging, Department of Radiology, Massachusetts General Hospital, Harvard Medical School, Charlestown, Massachusetts (USA)
Julie Price	Athinoula A. Martinos Center for Biomedical Imaging, Department of Radiology, Massachusetts General Hospital, Harvard Medical School, Charlestown, Massachusetts (USA)
Michael A. Levine	Athinoula A. Martinos Center for Biomedical Imaging, Department of Radiology, Massachusetts General Hospital, Harvard Medical School, Charlestown, Massachusetts (USA)
Michael Rullmann	Department of Nuclear Medicine, University of Leipzig, Leipzig (Germany)
Georg Alexander Becker	Department of Nuclear Medicine, University of Leipzig, Leipzig (Germany)
Henryk Barthel	Department of Nuclear Medicine, University of Leipzig, Leipzig (Germany)
Swen Hesse	Department of Nuclear Medicine, University of Leipzig, Leipzig (Germany)
Bernhard Sattler	Department of Nuclear Medicine, University of Leipzig, Leipzig (Germany)
Osama Sabri	Department of Nuclear Medicine, University of Leipzig, Leipzig (Germany)
Francesca Zanderigo	Department of Psychiatry, Columbia University, and the Molecular Imaging and Neuropathology Division, New York Psychiatric Institute, New York, NY (USA)
Harry Rubin-Falcone	Department of Psychiatry, Columbia University, and the Molecular Imaging and Neuropathology Division, New York Psychiatric Institute, New York, NY (USA)
Todd Ogden	Department of Psychiatry, Columbia University, and the Molecular Imaging and Neuropathology Division, New York Psychiatric Institute, New York, NY (USA)
Jarkko Johansson	Department of Radiation Sciences, Umeå University, S90187, Umeå (Sweden)
Lars Jonasson	Department of Radiation Sciences, Umeå University, S90187, Umeå (Sweden)
Filip Grill	Department of Radiation Sciences, Umeå University, S90187, Umeå (Sweden)
Nina Karalija	Department of Radiation Sciences, Umeå University, S90187, Umeå (Sweden)
Anna Rieckmann	Department of Radiation Sciences, Umeå University, S90187, Umeå (Sweden)
Ronald Boellaard	Department of Radiology and Nuclear Medicine, Amsterdam Neuroscience, Amsterdam UMC, Amsterdam (The Netherlands)
Sandeep Golla	Department of Radiology and Nuclear Medicine, Amsterdam Neuroscience, Amsterdam UMC, Amsterdam (The Netherlands)
Maqsood Yaqub	Department of Radiology and Nuclear Medicine, Amsterdam Neuroscience, Amsterdam UMC, Amsterdam (The Netherlands)
Kjell Erlandsson	Institute of Nuclear Medicine, University College London, London (UK)
Benjamin A. Thomas	Institute of Nuclear Medicine, University College London, London (UK)
Stefanie D. Krämer	Institute of Pharmaceutical Sciences, Department of Chemistry and Applied Biosciences, ETH Zurich, Zurich (Switzerland)
Lucas Narciso	Lawson Health Research Institute, London, Ontario (Canada)
Udunna Anazodo	Lawson Health Research Institute, London, Ontario (Canada)
Martin Norgaard	Neurobiology Research Unit & CIMBI, Copenhagen University Hospital, Rigshospitalet, Copenhagen (Denmark)
Melanie Ganz	Neurobiology Research Unit, Copenhagen University Hospital, Rigshospitalet, Copenhagen (Denmark) & Department of Computer Science, University of Copenhagen, Copenhagen (Denmark)
Martin Schain	Neurobiology Research Unit & CIMBI, Copenhagen University Hospital, Rigshospitalet, Copenhagen (Denmark)
Claus Svarer	Neurobiology Research Unit & CIMBI, Copenhagen University Hospital, Rigshospitalet, Copenhagen (Denmark)
Hanne D. Hansen	Neurobiology Research Unit & CIMBI, Copenhagen University Hospital, Rigshospitalet, Copenhagen (Denmark)
Gitte M. Knudsen	Neurobiology Research Unit & CIMBI, Copenhagen University Hospital, Rigshospitalet, Copenhagen (Denmark)
Christopher T. Smith	Department of Psychology, Vanderbilt University, Nashville, Tennessee (US)
My Jonasson	Radiology, Department of Surgical Sciences, Uppsala University, Uppsala (Sweden)
Mark Lubberink	Radiology, Department of Surgical Sciences, Uppsala University, Uppsala (Sweden)
Matteo Tonietto	Université Paris-Saclay, CEA, CNRS, Inserm, BioMaps, Service Hospitalier Frédéric Joliot, Orsay (France)

## Supplemental Material

sj-pdf-1-jcb-10.1177_0271678X211015101 - Supplemental material for Reproducibility of findings in modern PET neuroimaging: insight from the NRM2018 grand challenge Click here for additional data file. Supplemental material, sj-pdf-1-jcb-10.1177_0271678X211015101 for Reproducibility of findings in modern PET neuroimaging: insight from the NRM2018 grand challenge by Mattia Veronese, Gaia Rizzo, Martin Belzunce, Julia Schubert, Graham Searle, Alex Whittington, Ayla Mansur, Joel Dunn, Andrew Reader, Roger N Gunn and and the Grand Challenge Participants^#^ in Journal of Cerebral Blood Flow & Metabolism

sj-pdf-2-jcb-10.1177_0271678X211015101 - Supplemental material for Reproducibility of findings in modern PET neuroimaging: insight from the NRM2018 grand challenge Click here for additional data file. Supplemental material, sj-pdf-2-jcb-10.1177_0271678X211015101 for Reproducibility of findings in modern PET neuroimaging: insight from the NRM2018 grand challenge by Mattia Veronese, Gaia Rizzo, Martin Belzunce, Julia Schubert, Graham Searle, Alex Whittington, Ayla Mansur, Joel Dunn, Andrew Reader, Roger N Gunn and and the Grand Challenge Participants^#^ in Journal of Cerebral Blood Flow & Metabolism
