# Supplementary material for: Reproducibility of findings in modern PET neuroimaging: insight from the NRM2018 grand challenge
Source: J Cereb Blood Flow Metab. 2021 May 17;41(10):2778–96. doi: 10.1177/0271678X211015101 (PMC8504414; doi:10.1177/0271678X211015101)
Supplement: sj-pdf-1-jcb-10.1177_0271678X211015101 - Supplemental material for Reproducibility of findings in modern PET neuroimaging: insight from the NRM2018 grand challenge [file sj-pdf-1-jcb-10.1177_0271678X211015101.pdf]

Supplemental Table 1 - List of analysis methods and correspondent abbreviations

Method	***Definition***	***Reference***
LEGA with reference region	Likelihood Estimation in Graphical Analysis	[43]
Logan with reference region	Logan graphical method with tissue reference input function	[30]
MRTM	Multilinear Reference Tissue Model	[44]
MRTM2	Multilinear Reference Tissue Model with fixed k’2	[32]
SRTM	Simplified Reference Tissue Model	[20]
RPM	Basis function implementation for SRTM	[31]

Supplemental Table 2 - List of analysis software

Method	***Definition***
AFNI-3dClustSim	Program to estimate the probability of false positive (noise-only) clusters.
	WEB: https://afni.nimh.nih.gov/pub/dist/doc/program_help/3dClustSim.html
BrainFit	MATLAB-based pipeline software used in the Brain Imaging Lab in the Molecular Imaging and Neuropathology Division, Psychiatry, Columbia University and New York State Psychiatric Institute.
	WEB: Not publicly available
JIP	Neuroimaging analysis toolbox
	WEB: https://www.nitrc.org/projects/jip/
FreeSurfer	An open source software suite for processing and analyzing (human) brain MRI images
	WEB: https://surfer.nmr.mgh.harvard.edu
FSL	A library of analysis tools for FMRI, MRI and DTI brain imaging data
	WEB: https://fsl.fmrib.ox.ac.uk/fsl/fslwiki/
MATLAB	Mathworks ® - www.mathworks.com
MIAKAT™	A fully quantitative suite of analysis tools for PET neuroimaging data
	WEB: http://www.miakat.org
PETPVC	A toolbox for partial volume correction in positron emission tomography (PET)
	WEB: https://github.com/UCL/PETPVC
PMOD	Software for biomedical image quantification
	Brucker, Preclinical imaging division
	**WEB:** https://www.pmod.com/web/
PVElab	**Software for correction of functional images for partial volume errors**
	**WEB:** https://github.com/swederik/pvelab
SPM	**An open source software** for the **analysis of brain imaging data sequences**
	WEB: https://www.fil.ion.ucl.ac.uk/spm/
SnPM	A SPM toolbox for non parametric mapping
	WEB: http://www.nisox.org/Software/SnPM13/
