# Supplementary material for: Reproducibility of findings in modern PET neuroimaging: insight from the NRM2018 grand challenge
Source: J Cereb Blood Flow Metab. 2021 May 17;41(10):2778–96. doi: 10.1177/0271678X211015101 (PMC8504414; doi:10.1177/0271678X211015101)

Reproducibility of findings in modern PET neuroimaging: insight from the NRM2018 Grand Challenge

Supplementary Figure 1

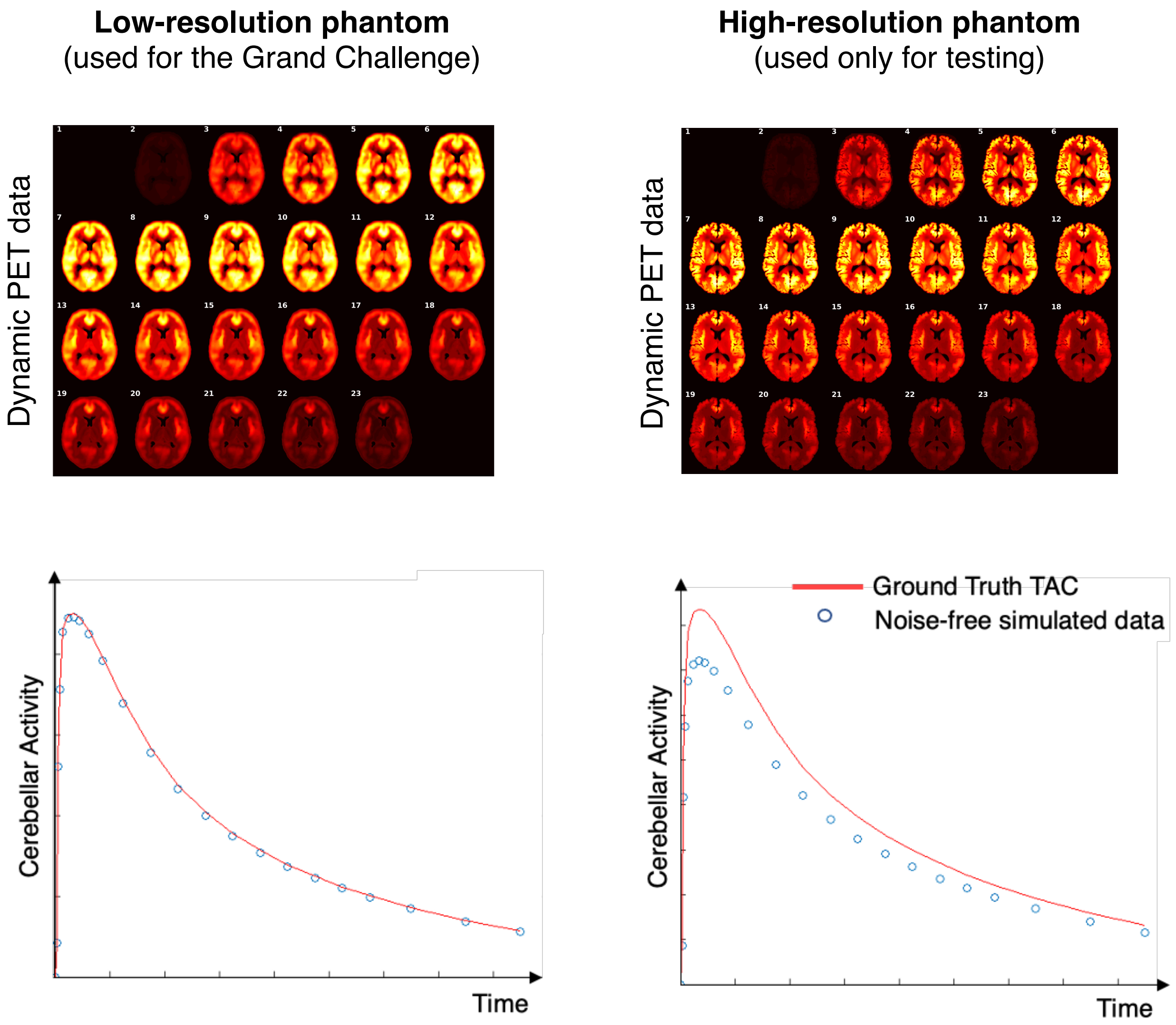

Supplement: sj-pdf-2-jcb-10.1177_0271678X211015101 - Supplemental material for Reproducibility of findings in modern PET neuroimaging: insight from the NRM2018 grand challenge [file sj-pdf-2-jcb-10.1177_0271678X211015101.pdf]
